# Miliary Nodules in the Lungs Not Always Due to Tuberculosis: A Report of a Rare Case

**DOI:** 10.7759/cureus.80120

**Published:** 2025-03-05

**Authors:** Archana Malik, Saurabh Karmakar, Suprova Chakraborty, Sarthak Das, Reshma Yadav

**Affiliations:** 1 Pulmonary Medicine, All India Institute of Medical Sciences, Deoghar, Deoghar, IND; 2 Pulmonary Medicine, All India Institute of Medical Sciences, Patna, Patna, IND; 3 Pediatrics, All India Institute of Medical Sciences, Deoghar, Deoghar, IND

**Keywords:** allergic bronchopulmonary aspergillosis (abpa), asthma, miliary opacity, misdiagnosed, pulmonary tuberculosis (ptb)

## Abstract

Allergic bronchopulmonary aspergillosis (ABPA) is a disease that occurs due to a pulmonary immune system reaction to the antigens of Aspergillus Fumigatus. Central bronchiectasis, mucoid impaction, mosaic attenuation, centrilobular nodules, and tree-in-bud opacities are the most commonly observed CT scan findings of ABPA. Miliary molting is one of the rare radiological manifestations of ABPA. ABPA is usually seen in uncontrolled asthma and is very uncommon in patients without a prior history of asthma. We are reporting a case of ABPA that presented a miliary nodule in radiology and was never diagnosed as a case of asthma previously. Thus, in a patient with eosinophilia and miliary molting, ABPA is to be excluded. Early diagnosis and treatment can prevent end-stage fibrotic ABPA.

## Introduction

Allergic bronchopulmonary aspergillosis (ABPA) is a pulmonary immune response to *Aspergillus fumigatus* antigens, primarily affecting individuals with asthma and cystic fibrosis. In India, the prevalence of ABPA among asthma patients is approximately 5% [1], while severe asthma cases show a significantly higher prevalence. A prospective study conducted at a tertiary care chest clinic in Northern India reported a prevalence of 70% among severe asthma patients [2].

High-resolution computed tomography (HRCT) of the thorax is the imaging modality of choice for diagnosing ABPA, with central bronchiectasis being the most common finding [3]. Other radiological features include mucoid impaction, mosaic attenuation, centrilobular nodules, and tree-in-bud opacities [3]. In a country like India, where tuberculosis (TB) is highly prevalent, ABPA is frequently misdiagnosed or diagnosed late. A study from Pakistan found that 82.5% of ABPA patients had previously received anti-tubercular treatment (ATT) before being correctly diagnosed with ABPA [4]. These cases were initially diagnosed based on chest X-rays alone [4].

Miliary opacity is a rare radiological manifestation of ABPA, with only four reported cases to date. Here, we present the fifth documented case of ABPA presenting with miliary opacity on a CT scan.

## Case presentation

**Figure 1:**
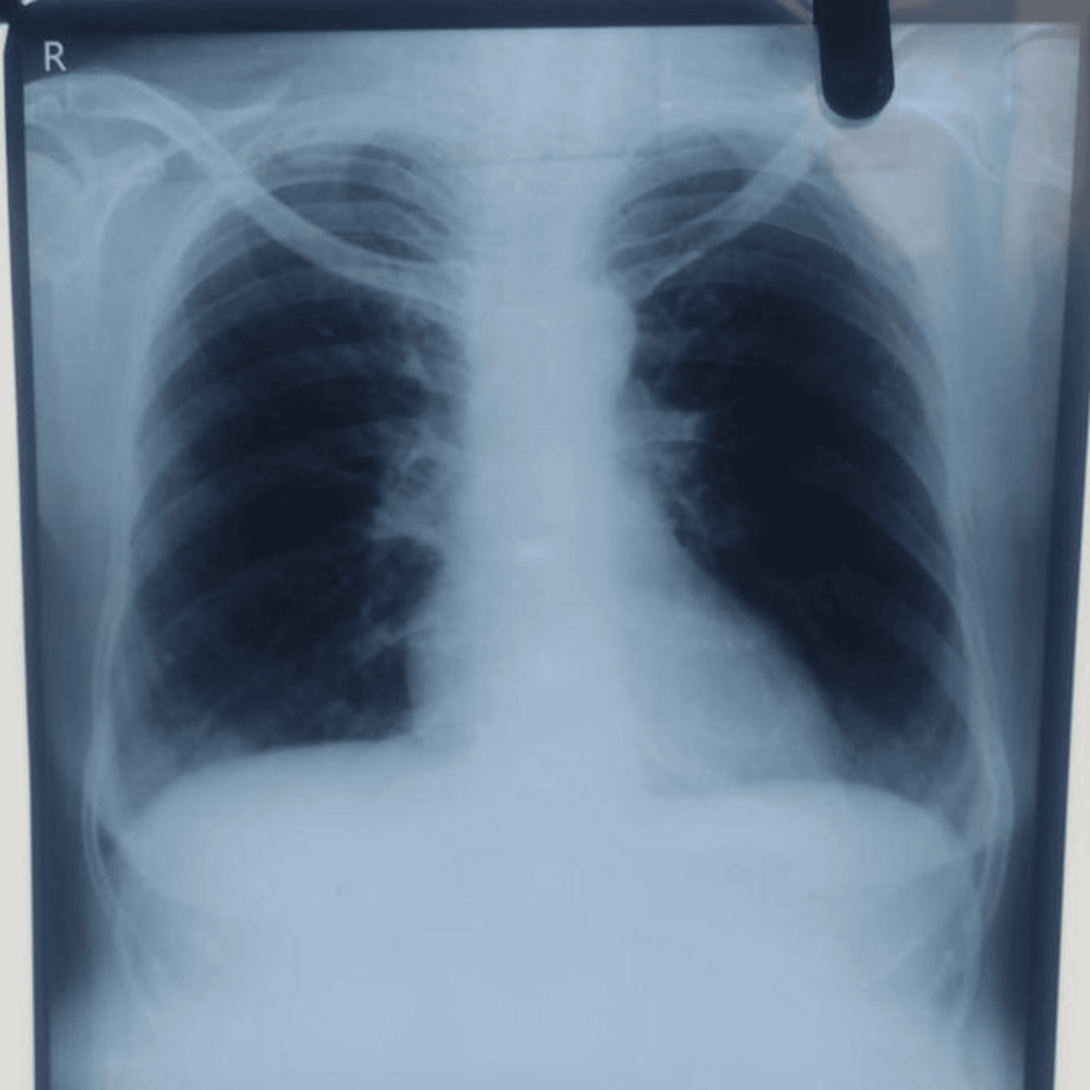
Normal chest X-ray posteroanterior (PA) view

A 45-year-old male, a businessman and non-smoker, presented to the Pulmonary Medicine outpatient department with complaints of breathlessness and dry cough for five days. The breathlessness was gradually progressive, classified as grade 2 on the Modified Medical Research Council (MMRC) dyspnea scale. There was no history of orthopnea, paroxysmal nocturnal dyspnea, pedal edema, or hemoptysis. Although he had no prior diagnosis of asthma, he reported intermittent wheezing and allergic rhinitis symptoms, particularly during winter, but was not on any medication. On examination, his respiratory rate was 16/min, pulse rate was 76/min, blood pressure was 100/80 mmHg in the right arm (supine position), and oxygen saturation by finger tip pulse oximetry was 97% on room air. General examination revealed no pallor, icterus, cyanosis, clubbing, pedal edema, raised JVP, or calf tenderness. Chest auscultation showed normal bilateral vesicular breath sounds with occasional rhonchi. A chest X-ray was normal (Figure 1), and laboratory findings showed a total leukocyte count of 12,000/mm³ with 34% neutrophils and 50% eosinophils, and the absolute eosinophil count was 1,600/mm³. Liver and kidney function tests were within normal limits, and RT-PCR for COVID-19 was negative. Spirometry revealed mild obstruction with significant bronchodilator reversibility, while ECG and echocardiography were unremarkable. He was initially treated for mild asthma exacerbation with a budesonide-formoterol inhaler (200 mcg, two puffs BD). However, after 10 days, his symptoms persisted, and he returned with tachypnea and mild wheezing. Given the discrepancy between symptoms and clinical signs, he was referred to psychiatry for possible anxiety and depression related to asthma, but his evaluation was normal. 

**Figure 2:**
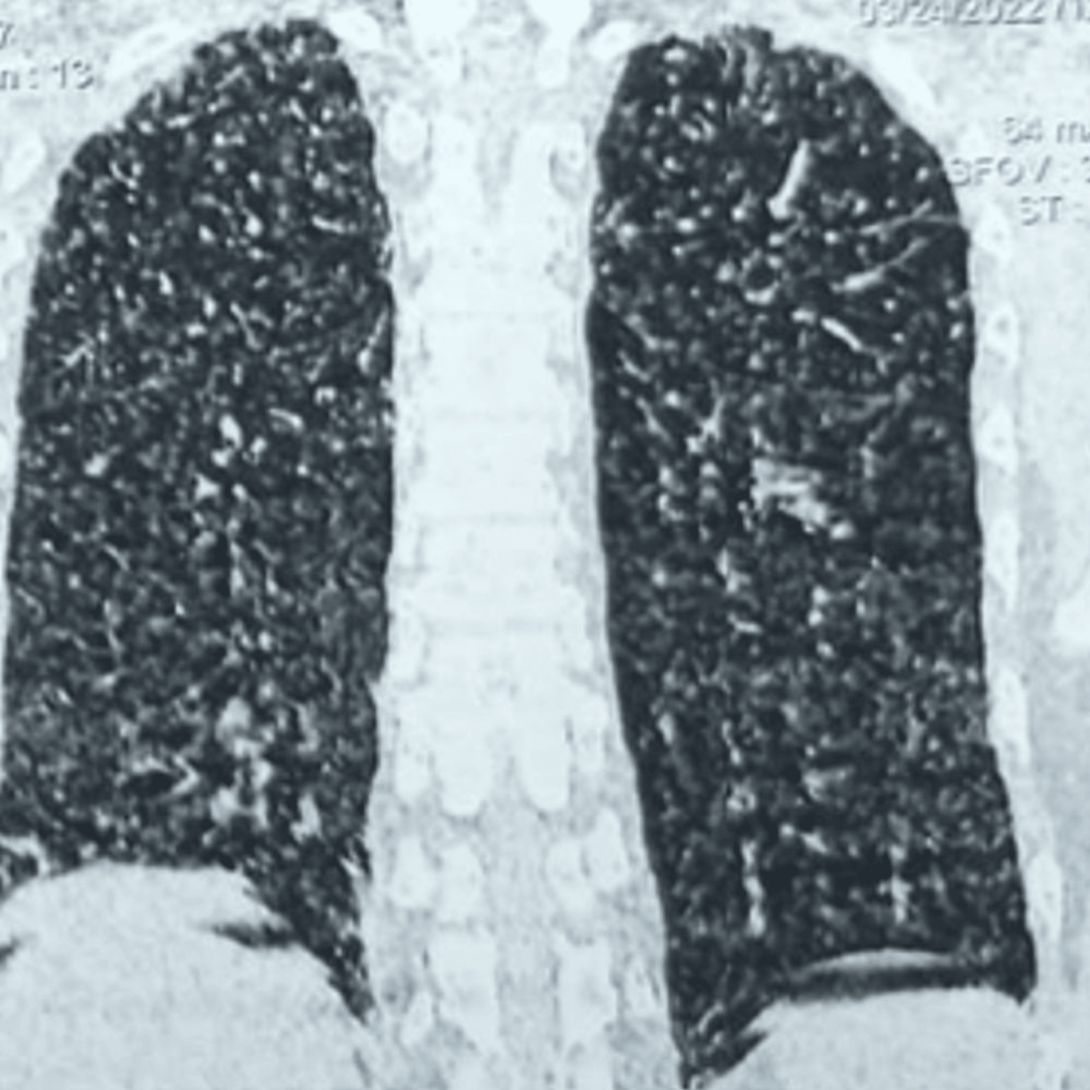
A coronal computed tomography of the lung window reveals opacity of size 1 to 3 mm all over the bilateral lungs (CT taken 12th day after X-ray)

**Figure 3:**
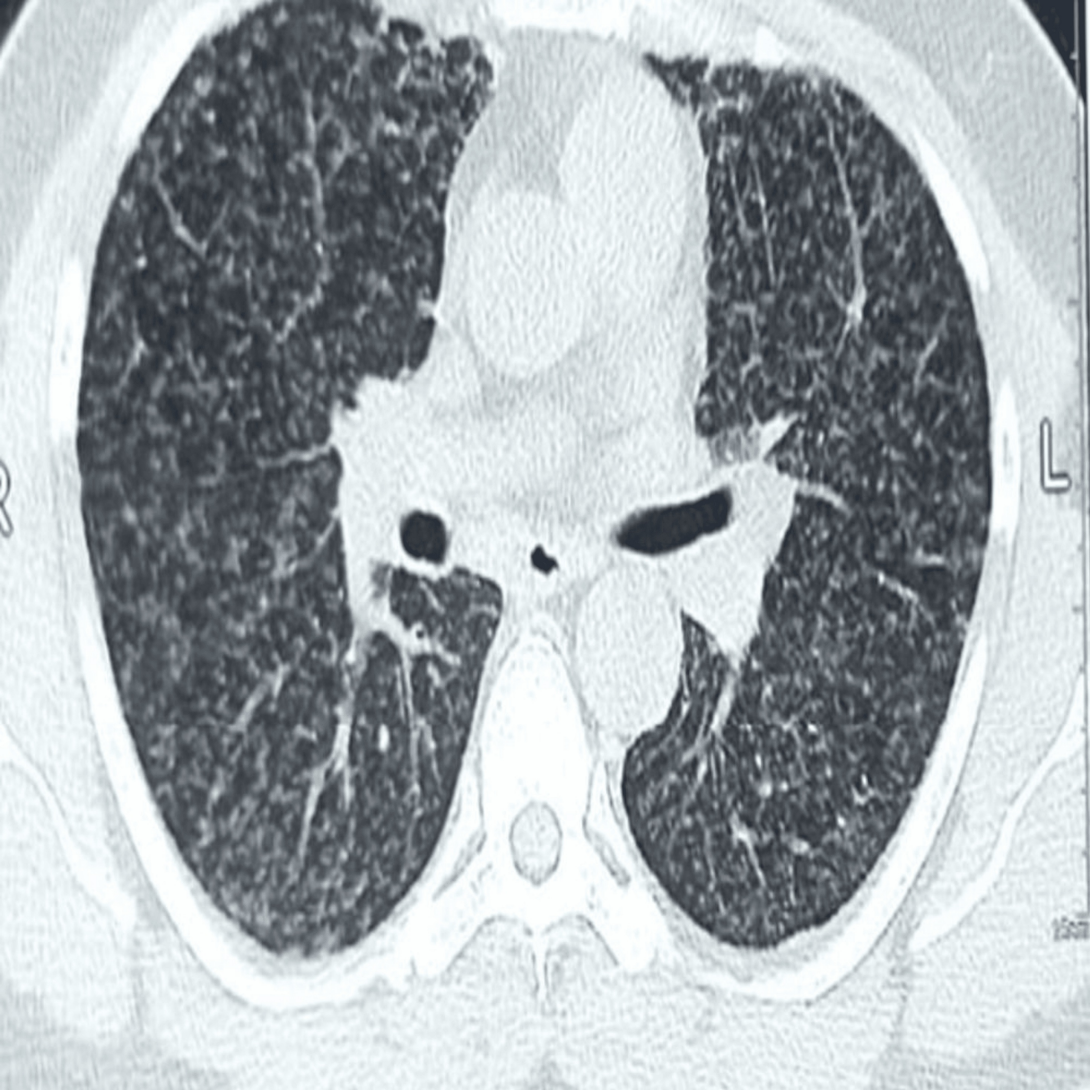
Transverse computed tomography of the lung window reveals a miliary nodular opacity all over the lung field

**Figure 4:**
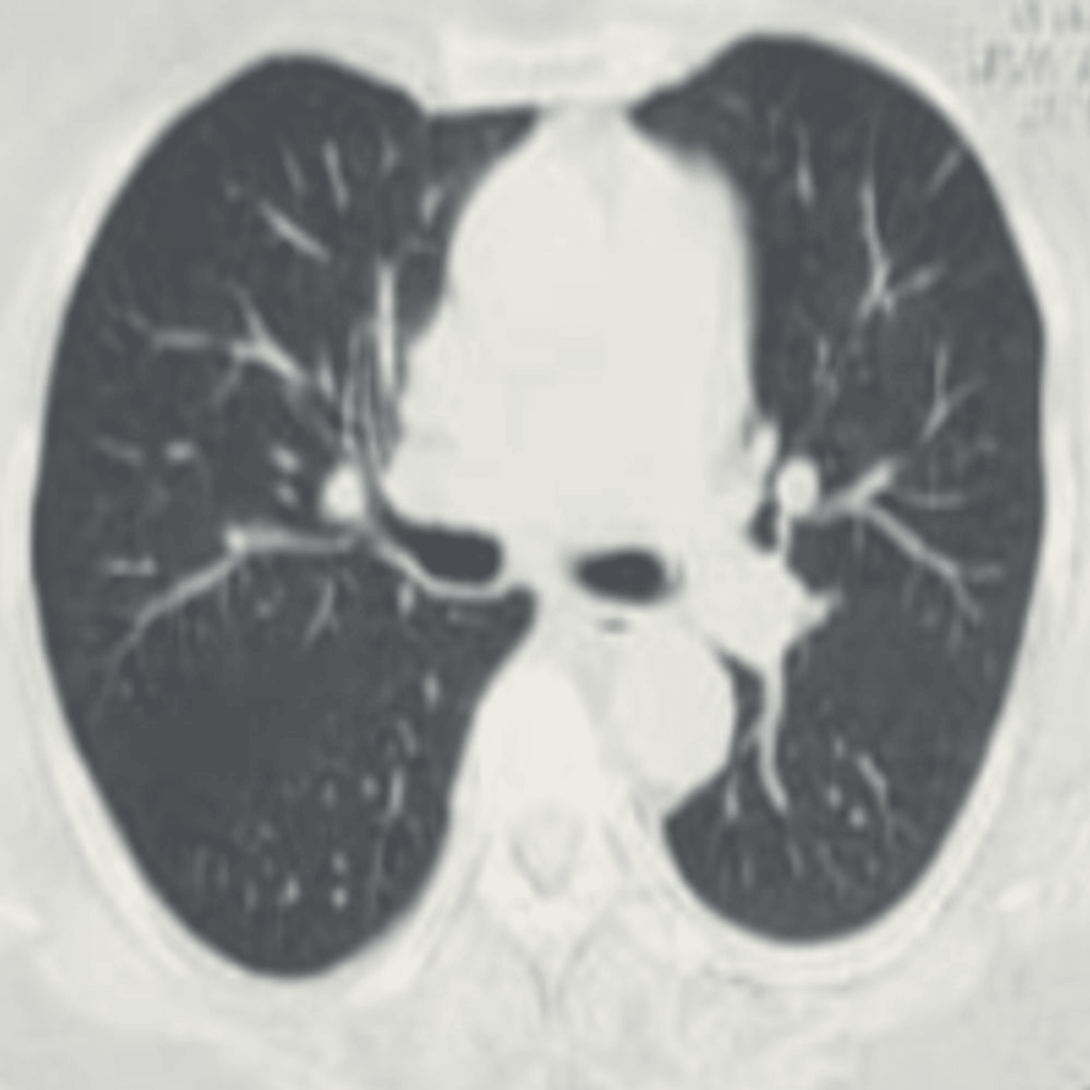
Transverse computed tomography of the lung window reveals at the end of course of steroid showing complete resolution of miliary opacity

Further investigations were conducted to rule out conditions such as subacute pulmonary embolism, eosinophilic granulomatosis with polyangiitis, interstitial lung disease, acute ABPA exacerbation, and tropical pulmonary eosinophilia. His D-dimer was 230 ng/mL, and both p-ANCA and c-ANCA were within normal limits. High-resolution CT (HRCT) of the thorax revealed multiple nodular opacities (1-3 mm) scattered across both lung fields, more prominent in the upper lobes, resembling miliary opacities ( Figures 2, 3, 4). No other interstitial abnormalities were noted. Mantoux test measured 5 mm, and induced sputum tests for AFB and CBNAAT were negative. A bronchoscopy was performed, revealing a normal tracheobronchial tree, and BAL CBNAAT was also negative. A transbronchial lung biopsy was conducted. Serological tests showed significantly elevated serum IgE (3,245 IU/mL), *Aspergillus*-specific IgE (0.87 IU/mL), and *Aspergillus*-specific IgG (240 mcg/mL). Ophthalmologic evaluation ruled out choroidal tubercles, and serum ACE and 24-hour urinary calcium levels were within normal limits. Based on the International Society for Human and Animal Mycology criteria, a final diagnosis of ABPA was established, and the patient was initiated on steroid therapy as per protocol. The transbronchial lung biopsy showed bronchial mucosa, with a few alveoli and no evidence of granuloma. He was kept on regular follow-up, and after completing a four-month tapering course of steroids, his HRCT thorax showed complete resolution of miliary opacities, with a reduction in serum IgE levels to 1,045 IU/mL.

A comprehensive table including all laboratory findings along with their reference ranges is presented in Table 1. 

**Table 1:** Clear and organized overview of all the diagnostic investigations performed

Investigation	Result	Reference range	Remarks
Chest X-ray	Normal	-	Figure 1
Total leukocyte count	12,000/mm^3^	4,000-11,000/mm3	Elevated
Neutrophils	34%	40-70%	Low
Eosinophils	50%	1-6%	Significantly elevated
Total eosinophil count	1,600/mm^3^	<500/mm^3^	Elevated
Liver function test	Within normal limits	-	Normal
Kidney function test	Within normal limits	-	Normal
Serum ACE level	Within normal limits	8-53 U/L	Sarcoidosis less likely
24-hour urinary calcium	Within normal limits	100-300 mg/day	Sarcoidosis less likely
RT-PCR for COVID-19	Negative	Negative	-
Mantoux test	5 mm	<10 mm	No strong evidence of TB
Induced sputum for AFB	Negative	Negative	TB ruled out
Induced sputum CBNAAT	Negative	Negative	TB ruled out
BAL CBNAAT	Negative	Negative	TB ruled out
Spirometry	Post bronchodilator FEV1/FVC=67%,FVC-82%(2.85L) FEV1-81%9(2.65L)and bronchodilator reversibility of 300 ml and 14%	FEV1/FVC < 70% (obstruction)	Consistent with asthma
D-dimer	230 ng/mL	<500 ng/mL	Normal
p-ANCA	Within normal limits	Negative	Autoimmune causes ruled out
c-ANCA	Within normal limits	Negative	Autoimmune causes ruled out
Serum IgE (initial)	3,245 IU/mL	<100 IU/mL	Significantly elevated
Serum IgE (after treatment)	1,045 IU/mL	<100 IU/mL	Decreased after steroids
Aspergillus-specific IgE	0.87 IU/mL	<0.35 IU/mL	Elevated
Aspergillus-specific IgG	240 mcg/mL	<50 mcg/mL	Elevated
HRCT thorax (initial) miliary	Miliary opacities (1–3 mm) predominantly in upper lobes	Normal lung fields should be clear	Figures 2, 3, 4
HRCT thorax (after treatment)	Complete resolution	-	Figure 4
Bronchoscopy	Normal tracheobronchial tree	-	No abnormalities detected
Transbronchial lung biopsy	No granuloma, no bronchial mucosa involvement	No granuloma, no bronchial mucosa involvement	Consistent with ABPA
Ophthalmologic evaluation	No choroidal tubercles	-	Sarcoidosis less likely

## Discussion

ABPA has been found to reveal underlying asthma and, in rare cases, present with miliary opacity on chest imaging. To date, only four reported cases have described ABPA with this radiological feature. Miliary opacity, characterized by numerous small nodules (<3 mm in diameter) scattered throughout both lungs, has a broad range of potential causes. These include infectious diseases such as miliary TB, histoplasmosis, mycoplasma, *Nocardia*, and blastomycosis; immune and inflammatory conditions like sarcoidosis, tropical pulmonary eosinophilia, and hypersensitivity pneumonitis; and malignant disorders such as bronchoalveolar carcinoma and hematogenous metastases from thyroid or kidney cancer, as well as lymphangitic carcinomatosis [5,6]. In endemic regions like India, TB remains the leading cause of miliary opacity on chest CT. A definitive diagnosis requires microbiological testing, including sputum analysis, bronchoalveolar lavage, and transbronchial lung biopsy. However, in resource-limited settings, miliary TB is often diagnosed based on CT imaging alone, leading to frequent misdiagnosis.

ABPA is a hypersensitivity reaction to the *Aspergillus* species, primarily *Aspergillus fumigatus*. It significantly affects asthma control, contributing to poor disease management and increased morbidity. Patients with ABPA frequently experience chronic mucus hypersecretion, a known risk factor for accelerated lung function decline [7]. Common symptoms include hemoptysis, low-grade fever, weight loss, malaise, and expectoration of brownish mucus plugs, a characteristic finding in ABPA [8]. The condition is most commonly observed in individuals with uncontrolled asthma. In India, asthma prevalence among adults is estimated at 27.6%, with the burden of ABPA ranging from 0.12 to 6.09 million cases [9]. The prevalence of ABPA among asthma patients is approximately 13%, and the incidence is even higher in severe asthma cases. Around 70% of patients with severe asthma exhibit sensitization to various fungi [10,11]. Notably, in our case, the patient had no prior asthma diagnosis, and ABPA led to its clinical identification.

The clinical presentation of ABPA is often non-specific, including chronic cough, wheezing, worsening asthma, and recurrent pulmonary infiltrates [12]. By contrast, miliary TB is typically characterized by prolonged fever, anorexia, weight loss, malaise, and persistent cough [13]. A key distinguishing factor is fever, which is common in TB but rare in ABPA. HRCT of the thorax is the imaging modality of choice for ABPA, with high-attenuation mucus (HAM) being a pathognomonic finding [14]. However, ABPA may also present without radiological abnormalities. While CT findings can aid classification, they are not mandatory for diagnosis. Conversely, a normal CT scan effectively rules out miliary TB, as imaging is diagnostic in such cases.

The diagnosis of ABPA follows the International Society for Human and Animal Mycology (ISHAM) Working Group criteria [15]. It requires a predisposing condition (asthma) and the presence of two obligatory criteria: (1) serum IgE levels ≥1000 IU/mL and (2) a positive skin prick test for *Aspergillus* or elevated *Aspergillus*-specific IgE. In addition, at least two out of three supportive criteria must be met: (1) eosinophil count >500/mm³, (2) elevated *Aspergillus*-specific IgG, and (3) consistent radiological features. By contrast, there are no definitive diagnostic criteria for miliary opacity, and TB is generally diagnosed by exclusion. Supporting evidence includes extrapulmonary manifestations such as choroidal tubercles in the eye or miliary lesions in the liver and spleen. Further confirmation is obtained through transbronchial or cryobiopsy of the lung.

The primary treatment for ABPA involves systemic corticosteroids, whereas miliary TB requires anti-tubercular therapy. The difference between miliary TB and ABPA is presented with miliary opacity (Table 2).

**Table 2:** Difference between miliary tuberculosis and allergic bronchopulmonary opacity presented with miliary opacity

	Military tuberculosis	ABPA
Underlying condition	Immune suppressed condition like old age, diabetes, chronic kidney disease, chronic liver disease	Asthma, cystic fibrosis, uncommonly COPD
Clinical feature	Fever is common	Fever with evening rise in temperature is unusual
Montoux test	Positive	Negative
Peripheral eosinophilia	Usually absent	Usually present(obligatory criteria)
HRCT thorax	Normal - ruled out the probability	May be normal proven serologically
Treatment	ATT	Steroid VVV

## Conclusions

TB is the most common cause of miliary opacity in CT in India. In areas with a high prevalence of PTB, ABPA is often misdiagnosed. Patients with ABPA having atypical radiological presentations are often misdiagnosed with PTB. ABPA cases misdiagnosed as PTB in India are as high as 17-50% (17). When the patient with miliary molting in a CT scan presented with eosinophilia and without fever, ABPA can be kept as a differential diagnosis. Based on miliary opacity in a CT scan, it will be unwise to give ATT to the patient. Early diagnosis and management of ABPA in such cases will help prevent the development of end-stage pulmonary fibrosis. Moreover, a careful evaluation of patients with miliary opacity can prevent unnecessary ATT prescriptions.
